# Increased thalamic centrality and putamen–thalamic connectivity in patients with parkinsonian resting tremor

**DOI:** 10.1002/brb3.601

**Published:** 2016-11-23

**Authors:** Quanquan Gu, Hengyi Cao, Min Xuan, Wei Luo, Xiaojun Guan, Jingjing Xu, Peiyu Huang, Minming Zhang, Xiaojun Xu

**Affiliations:** ^1^ Department of RadiologyThe Second Affiliated HospitalZhejiang University School of MedicineHangzhouChina; ^2^ Department of Psychiatry and PsychotherapyCentral Institute of Mental HealthUniversity of Heidelberg Medical Faculty MannheimMannheimGermany; ^3^ Department of NeurologyThe Second Affiliated HospitalZhejiang University School of MedicineHangzhouChina

**Keywords:** functional connectivity, functional MRI, graph theory, Parkinson's disease, resting tremor

## Abstract

**Introduction:**

Evidence has indicated a strong association between hyperactivity in the cerebello‐thalamo‐motor cortical loop and resting tremor in Parkinson's disease (PD). Within this loop, the thalamus serves as a central hub based on its structural centrality in the generation of resting tremor. To study whether this thalamic abnormality leads to an alteration at the whole‐brain level, our study investigated the role of the thalamus in patients with parkinsonian resting tremor in a large‐scale brain network context.

**Methods:**

Forty‐one patients with PD (22 with resting tremor, TP and 19 without resting tremor, NTP) and 45 healthy controls (HC) were included in this resting‐state functional MRI study. Graph theory‐based network analysis was performed to examine the centrality measures of bilateral thalami across the three groups. To further provide evidence to the central role of the thalamus in parkinsonian resting tremor, the seed‐based functional connectivity analysis was then used to quantify the functional interactions between the basal ganglia and the thalamus.

**Results:**

Compared with the HC group, patients with the TP group exhibited increased degree centrality (*p *< .04), betweenness centrality (*p *< .01), and participation coefficient (*p *< .01) in the bilateral thalami. Two of these alterations (degree centrality and participation coefficient) were significantly correlated with tremor severity, especially in the left hemisphere (*p *< .02). The modular analysis showed that the TP group had more intermodular connections between the thalamus and the regions within the cerebello‐thalamo‐motor cortical loop. Furthermore, the data revealed significantly enhanced functional connectivity between the putamen and the thalamus in the TP group (*p *= .027 corrected for family‐wise error).

**Conclusions:**

These findings suggest increased thalamic centrality as a potential tremor‐specific imaging measure for PD, and provide evidence for the altered putamen–thalamic interaction in patients with resting tremor.

## 1 Introduction

Parkinson's disease (PD) is characterized by varying degrees of cardinal motor symptoms, including resting tremor, bradykinesia, and rigidity (Lees, Hardy, & Revesz, 2009). Resting tremor occurs in three of every four patients with PD (Hughes, Daniel, Blankson, & Lees, 1993; Mittel, 2003), but the neural mechanisms underlying resting tremor are still unclear.

Previous studies have shown that the “hyperactivity” in the cerebello‐thalamo‐motor cortical (CRB‐THA‐MC) loop significantly correlates to the generation of parkinsonian resting tremor (Deiber et al., 1993; Pollok et al., 2009; Timmermann et al., 2003). Recent studies further identified that enhanced interactions between the CRB‐THA‐MC loop and the basal ganglia, and in particular the putamen and pallidum, contribute to the generation of resting tremor (Duval, Daneault, Hutchison, & Sadikot, 2015; Helmich, Janssen, Oyen, Bloem, & Toni, 2011). Within the CRB‐THA‐MC loop, the thalamus serves as a pivotal node based on its structural centrality. Specifically, the thalamus is the gateway to multiple cortical sensory regions that facilitates sensory movement integration via the corticothalamic interaction (Le Masson, Renaud‐Le Masson, Debay, & Bal, 2002), and also relays cerebellar fibers projecting to cortical regions to mediate voluntary movements via the cerebellothalamic interaction (Aumann, 2002). The thalamus is considered a tremor pacemaker in PD (Cagnan et al., 2014; Duval et al., 2015); moreover, evidence has shown that dysfunctions in both the corticothalamic (Pollok, Gross, Dirks, Timmermann, & Schnitzler, 2004; Schnitzler & Gross, 2005) and cerebellothalamic (Helmich, Janssen, et al., 2011) interactions are potential contributors to tremor symptoms. By modulating the regional activity of the CRB‐THA‐MC loop (Fukuda et al., 2004), the thalamic deep brain stimulation (DBS) has obtained satisfactory outcomes in the treatment of resting tremor (Mehanna, Machado, Oravivattanakul, Genc, & Cooper, 2014; Rehncrona et al., 2003). These findings suggest that resting tremor is highly related to the abnormality in the thalamus‐centered CRB‐THA‐MC loop and the aberrant interactions between this loop and the basal ganglia.

On the basis of the above evidence, we speculated that (1) given the hyperactivity state of the thalamic function, parkinsonian resting tremor may be associated with higher thalamic centrality in the whole‐brain network, and (2) an enhanced interaction between the thalamus and the basal ganglia may be present in patients with parkinsonian resting tremor. Here, we tested these hypotheses in two PD subgroups characterized by the presence or absence of resting tremor. For the first hypothesis, graph theory‐based network analysis was performed, and the centrality measures of the bilateral thalami were examined. For the second hypothesis, seed‐based functional connectivity analysis was used to quantify the functional interactions between the basal ganglia and the thalamus.

## 2 Subjects and Methods

### 2.1 Participants

Forty‐one patients with PD (aged 57.76 ± 7.94 years, 25 males) and 45 age‐ and sex‐matched healthy controls (HC) (aged 56.98 ± 10.02 years, 24 males) participated in the study. The patients were recruited from the Department of Neurology, The Second Affiliated Hospital of Zhejiang University, and the controls were recruited in and around Hangzhou, China. All participants provided written informed consent for the protocols, which were approved by the institutional review board at Zhejiang University School of Medicine. The diagnoses of PD were based on disease history, clinical examinations, scale evaluations, response to dopaminergic medications, and exclusion of other neurologic and psychiatric diseases, according to the UK Parkinson's Disease Society Brain Bank criteria. While off medication, all patients were given a battery of clinical tests, including the UPDRS (Goetz et al., 2007), the Hoehn and Yahr scale (Hoehn & Yahr, 1967), the Mini‐Mental State Examination (MMSE) (Folstein, Folstein, & McHugh, 1975), and the Hamilton Depression Rating Scale (HDRS) (Hamilton, 1960). Twenty‐three patients were taking stable dopaminergic medications, and 18 patients had received no treatment prior to the date that the scans were performed. Of the participating patients, 22 had resting tremor (TP group, aged 58.82 ± 7.56 years, 15 males, 8 drug‐naive), and 19 did not have resting tremor (NTP group, aged 56.53 ± 8.38 years, 10 males, 10 drug‐naive).

The inclusion criteria for the two patient groups were based on the UPDRS resting tremor score (item 20), where patients in the TP group were defined by the presence of resting tremor at the head–neck region or at least one extremity, and patients in the NTP group were defined by the absence of resting tremor. Here, we carefully matched for other potential confounds between the two groups, including illness durations, general disease severity, rigidity, and bradykinesia scores (item 22 and items 23–26, respectively), general cognitive and affective assessments, and levodopa equivalent medication daily dose (LEDD) (for details, see Table 1). The exclusion criteria for healthy controls included a self‐reported history of neurological or psychiatric illness, prior drug or alcohol abuse, or head trauma (Table 1).

**Table 1:** Demographic and clinical characteristics for the participants

	Patients with resting tremor (*n *= 22)	Patients without resting tremor (*n *= 19)	Healthy controls (*n *= 45)	*p* value
Age (year)	58.82 ± 7.56	56.53 ± 8.38	56.98 ± 10.02	.67
Sex (M/F)	15/7	10/9	24/21	.47
Illness duration (year)	5.56 ± 5.06	3.57 ± 3.00	–	.13
Hoehn and Yahr scale	2.27 ± 0.72	2.45 ± 0.64	–	.42
UPDRS	42.55 ± 21.44	37.63 ± 17.12	–	.43
Part I	1.59 ± 2.44	1.89 ± 2.47	–	.70
Part II	9.50 ± 4.80	10.00 ± 4.92	–	.74
Part III	29.73 ± 15.52	24.89 ± 12.89	–	.29
Part IV	1.73 ± 2.21	0.84 ± 1.95	–	.19
Tremor score	8.82 ± 5.47	1.26 ± 1.28	–	<.0001^a^
Resting tremor	4.95 ± 3.55	0.00 ± 0.00	–	<.0001^a^
Action/posture tremor	2.23 ± 1.54	0.74 ± 0.93	–	.001^a^
Rigidity score	5.68 ± 4.38	7.31 ± 5.36	–	.29
Bradykinesia score	11.95 ± 7.22	11.47 ± 6.14	–	.82
MMSE	28.00 ± 1.63	27.58 ± 1.77	–	.43
HDRS	6.45 ± 9.05	9.05 ± 8.90	–	.36
Treatment status (under medication/drug‐naive)	14/8	9/10	–	.30
LEDD (mg)	389.7 ± 416.55	274.6 ± 362.85	–	.36
Head motion translation (mm)	0.50 ± 0.33	0.44 ± 0.27	0.49 ± 0.36	.78
Head motion rotation (degree)	0.57 ± 0.52	0.45 ± 0.25	0.45 ± 0.36	.45
Head motion framewise displacement (mm)	0.07 ± 0.03	0.08 ± 0.05	0.09 ± 0.04	.17

UPDRS, Unified Parkinson's Disease Rating Scale; MMSE, Mini‐Mental State Examination; HDRS, Hamilton Depression Rating Scale; LEDD, Levodopa Equivalent Daily Dose, calculated according to the method in Tomlinson et al. (2010).

a Significant *p* values (*p *< .05).

### 2.2 Data acquisition

Resting‐state functional magnetic resonance imaging (fMRI) data were acquired using a 3T GE Signa EXCITE scanner (GE Healthcare, Milwaukee, WI, USA) with a gradient‐recalled echo planar imaging (GRE‐EPI) sequence as follows: TR = 2000 ms, TE = 30 ms, matrix = 64 × 64, FOV = 24 × 24 cm^2^, flip angle = 80º, 23 slices, and 5 mm slice thickness. The whole scan lasted approximately 6.2 min (185 time points). During the scans, the participants were asked to lie still in the scanner, close their eyes, and not to engage in any particular mental activity. After the scans, the experimenter confirmed with each subject that they did not fall asleep during the scan.

### 2.3 Data preprocessing and head motion check

The data preprocessing followed the standard procedures implemented in the Statistical Parametric Mapping package (SPM8, http://www.fil.ion.ucl.ac.uk/spm/software/spm8, RRID:SCR_007037). The first 10 time points for each subject were discarded due to instability of the initial MR signals. The rest of the time series was slice‐timing–corrected to the middle slice, corrected for head motion, and normalized to the Montreal Neurological Institute (MNI) brain space with a resampled voxel size of 3 × 3 × 3 mm^3^. Finally, all images were smoothed with a 9‐mm full‐width at half‐maximum (FWHM) Gaussian kernel.

We quantified three head motion parameters that were used in previous studies (Plichta et al., 2012; Satterthwaite et al., 2013): the sum of the volume‐to‐volume translational excursions, the sum of the volume‐to‐volume rotational excursions, and the voxel‐level frame‐wise displacement. The first two measures calculate the sum of the root mean square of three translational and rotational motion vectors from the *x*,* y*, and *z* axes, respectively. The third measure is a nonlinear combination of volume‐wise translations and rotations, reflecting the voxel‐specific distance compared to the previous image. Details of these measurements are described in previous literature (Plichta et al., 2012; Satterthwaite et al., 2013). For the purpose of quality control, we carefully checked several head motion parameters for each subject. We confirmed that there were no between‐group differences in any of the calculated head motion parameters (all *p*s > 0.17, Table 1).

### 2.4 Brain graph analysis

#### 2.4.1 Construction of functional brain graphs

Our first hypothesis was tested by graph theory‐based brain network analysis. The entire procedure of graph analysis followed our previously published studies (Cao et al., 2014, 2016). Here, nodes were defined as the 92 anatomical brain regions (including the bilateral cerebellum) derived from the Automated Anatomical Labeling (AAL, RRID:SCR_003550) brain atlas (Tzourio‐Mazoyer et al., 2002), and links were computed with Pearson correlations. The graph analysis started with the extraction of the averaged time series from each of the 92 nodes. These raw time series were further corrected for white matter (WM) signal, cerebrospinal fluid (CSF) signal, and head motion nuisances. Because head motion is a potential detriment to graph analysis, which may induce spurious correlation estimates (Power, Barnes, Snyder, Schlaggar, & Petersen, 2012), we adopted a strict motion removal method, as reported in a previous study (Satterthwaite et al., 2013), to maximally reduce its influence on our results. Specifically, 24 head motion nuisances (i.e., 6 rigid‐body parameters generated from the realignment step, their first derivatives, and the squares of these 12 parameters) were calculated and regressed out from the time series. The residual time series were band‐pass filtered with 0.01–0.08 Hz. The whole‐brain connectivity matrices were subsequently computed by the pairwise correlations between the processed time series of each of the 92 nodes. Briefly, a 92 × 92 pairwise correlation matrix was calculated for each subject and subsequently was thresholded into a series of binary adjacency matrices.

To construct brain networks, the connectivity matrices were further thresholded into 31 densities ranging from 0.10 to 0.40 with an interval of 0.01. The density range was chosen based on previous empirical data showing that small‐world networks are retained within the same range during the resting state (Braun et al., 2012; Cao et al., 2014, 2016). In each density, a value of 1 (connected) was assigned to entries that survived the threshold, and a value of 0 (not connected) was assigned to those did not, resulting in a 92 × 92 binary matrix for each of the 31 densities for each subject. The centrality measures of the a priori nodes were subsequently computed from each of the binary matrices.

#### 2.4.2 Calculation of node centrality

The centrality measures typically reflect the importance of a given brain region in the whole‐brain network. We performed the calculation of node centrality using the Brain Connectivity Toolbox (https://sites.google.com/site/bctnet/, RRID:SCR_004841). Because our hypothesis focused on the thalamus, we primarily examined the centrality measures for bilateral thalami. We also calculated the same measures for bilateral cerebella and bilateral primary motor cortices as an investigation of nodal specificity because these regions are also involved in the CRB‐THA‐MC loop. Specifically, four commonly used centrality metrics for the above regions were calculated: degree centrality, betweenness centrality, within‐module degree, and participation coefficient (Buckner et al., 2009; Bullmore & Bassett, 2011; He et al., 2009; van den Heuvel & Sporns, 2013; Meunier, Achard, Morcom, & Bullmore, 2009; Power, Schlaggar, Lessov‐Schlaggar, & Petersen, 2013; Rubinov & Sporns, 2010; Zuo et al., 2012). The first two measures assess the importance of a given node from a more global perspective, and the last two probe the nodal centrality at a more modular level, thereby providing complementary information for a detailed description of a node's role in a complex network. In the following section, we briefly summarize the concepts and computations of these centrality measures. For more details, see related studies described previously (Bullmore & Bassett, 2011; van den Heuvel & Sporns, 2013; Power et al., 2013; Rubinov & Sporns, 2010; Zuo et al., 2012).

### 2.5 Centrality measures

#### 2.5.1 Degree and betweenness centrality

Degree centrality quantifies the total number of links in a network that are connected to a given node. Mathematically, it can be presented as the formula below:

$$K_{i}=\sum_{j=1}^{N}a_{ij}$$

where $K_{i}$ is the degree of node *i* and $a_{ij}$ is the connection between nodes *i* and *j*. A large value of degree centrality generally reflects the high importance of a given node in a network (Bullmore & Bassett, 2011; van den Heuvel & Sporns, 2013; Rubinov & Sporns, 2010).

Betweenness centrality is defined as the summed proportion of all shortest paths in a network that pass through a given node, given by the following formula:

$$B_{i}=\sum_{h\neq j\neq i}\frac{\sigma_{hj}\left(i\right)}{\sigma_{hj}}$$

where $B_{i}$ is the betweenness of node *i* and $\sigma_{hj}$ is the number of the shortest paths between nodes *h* and *j*. $\sigma_{hj}\left(i\right)$ is the number of the shortest paths between nodes *h* and *j* that pass through node *i*. A node with high betweenness centrality has a large influence on the transfer of information through the network, under the assumption that information transfer follows the shortest path (Buckner et al., 2009; Bullmore & Bassett, 2011; Rubinov & Sporns, 2010).

#### 2.5.2 Within‐module degree and participation coefficient

Despite their broad applications, degree and betweenness centrality metrics both bear the limitation that they are easily confounded by the embedded network structures, such as community size (Power et al., 2013). This limitation complicates the interpretation of the outcomes, leading to an elusive delineation of the precise role of a given node in the network and, accordingly, how its role changes with the change in its values. As a result, two module‐based measures, namely the within‐module degree and the participation coefficient, were proposed for a detailed role definition, which illustrates how a node is positioned in its own module and with respect to other modules (Guimera & Nunes Amaral, 2005). These measures can be computed provided that the maximal modular partition of a network has been identified.

For the modular partitions, the modularity metrics were calculated for each subject and demonstrate the degree to which a given network can be divided into nonoverlapping modules (Newman, 2004). The optimal modular structures were achieved by maximizing the modularity metrics, which were estimated heuristically according to the optimization algorithm proposed in a previous study (Newman, 2006). This optimization procedure was repeated 100 times for each subject, and the maximal values over these optimizations were acquired.

Within‐module degree is a measure of normalized local degree centrality of a given node. It reflects the relative importance of the given node compared to the other nodes in the same module and is expressed mathematically as:

$$Z_{i}=\frac{K_{i}\left(M_{i}\right)-\overset{¯}{K}\left(M_{i}\right)}{\sigma^{K(M_{i})}}$$

where $Z_{i}$ is the within‐module degree of node *i*, $K_{i}\left(M_{i}\right)$ is the number of links between node *i* and all other nodes in the module $M_{i}$. $\overset{¯}{K}\left(M_{i}\right)$ and $\sigma^{K(M_{i})}$ are the mean and standard deviation of the degree distribution of module $M_{i}$. A large value of *Z* indicates a large number of intramodular connections relative to the other nodes in the same module (Guimera & Nunes Amaral, 2005; He et al., 2009; Meunier et al., 2009; Rubinov & Sporns, 2010).

Accordingly, a measure for intermodular connections of a given node is defined by a participation coefficient:

$$P_{i}=1-\sum_{m=1}^{N(m)}{\left(\frac{K_{i}\left(m\right)}{K_{i}}\right)}^{2}$$

where $P_{i}$ is the participation coefficient of node *i* and $K_{i}\left(m\right)$ is the number of links between *i* and all other nodes in the given module *m*. *N* (*m*) is the number of the all modules in the network. This measure quantifies the ability of a given node in connecting different modules. $P_{i}$ has a maximal value (close to 1) if its connections are uniformly distributed among all modules and a value of 0 if it is exclusively connected to the nodes within its own module (Guimera & Nunes Amaral, 2005; He et al., 2009; Meunier et al., 2009; Power et al., 2013; Rubinov & Sporns, 2010).

### 2.6 Statistics

Statistical analysis was performed with the SPSS 20 software (IBM SPSS, Chicago, IL, USA, RRID:SCR_002865). Here, the measures of centrality for each of the six examined nodes were entered as dependent variables into a repeated‐measures analysis of covariance (ANCOVA) model, where densities were included as within‐subject factors and groups (TP, NTP, HC) were included as between‐subject factors. Age and sex were also set as covariates of noninterest. Of note, measures of within‐module degree and participation coefficient are dependent on optimal modularity estimates; therefore, we also examined the group differences in modularity to ensure that the centrality differences were not confounded by the deviations in modular partition qualities. Because the measured graph metrics are highly interdependent (Cao et al., 2014; Lynall et al., 2010) and our primary hypothesis relates to the bilateral thalami, no specific multiple corrections were needed. Statistical significance was thus set at *p *< .05.

For the altered graph metrics identified in the TP group, we further investigated whether changes in these measures were associated with tremor severity. Specifically, we performed Pearson partial correlations between the centrality measures (averaged across densities) and the UPDRS tremor scores (resting tremor scores and total tremor scores) for the two patient groups separately, adjusting for age and sex. Resting tremor scores were defined as UPDRS item 20, and total tremor scores were defined as the sum of UPDRS items 16 (tremor history), 20 (tremor at rest), and 21 (action/posture tremor). To test the specificity of the correlation findings, we further examined partial correlations between these graph measures and the UPDRS bradykinesia subscores (the sum of UPDRS items 23–26), the UPDRS rigidity scores (UPDRS item 22), the overall UPDRS scores, and the UPDRS subscores for each part.

### 2.7 Seed connectivity analysis

Seed‐based functional connectivity analysis was used to test our second hypothesis. In accordance with the findings from Helmich, Janssen, et al. (2011), two seed regions were defined using the AAL brain template: the putamen and the pallidum (combining both hemispheres). The mean time series of each of the two seeds (i.e., the putamen and pallidum) were extracted from the preprocessed data, corrected for WM and CSF signals and head motions, and entered separately into an individual first‐level general linear model (GLM) implemented in SPM8 as a regressor of interest. The following regressors were also included in the model as variables of noninterest: WM signal, CSF signal, and the 24 head motion nuisances derived from the graph analysis. The estimated first‐level beta images were further used for a second‐level random effects analysis with the groups (TP, NTP, HC) as the independent variable and age and sex as covariates. To focus on our second hypothesis, an a priori anatomical mask of bilateral thalami was created from the AAL template, and the results were reported after performing small‐volume family‐wise error (FWE) correction across the mask. Similar to the procedures in the graph analysis, we further tested the regional specificity of the connectivity findings using masks of the bilateral cerebella and bilateral primary motor cortices. Between‐group differences across these masks were also investigated.

To examine the clinical associations of the altered connectivity identified in patient groups, we further extracted subject‐specific eigenvariates of the connectivity measures (beta estimates) from 6‐mm radius spheres centered at each of the peak voxels derived from the group‐wise comparisons. For each patient group, Pearson partial correlation coefficients were calculated between these measures and the UPDRS scores (tremor scores, bradykinesia scores, rigidity scores, overall scores and subscores for each part), adjusting for age and sex.

## 3 Results

### 3.1 Alterations in nodal centrality

**Figure 1:**
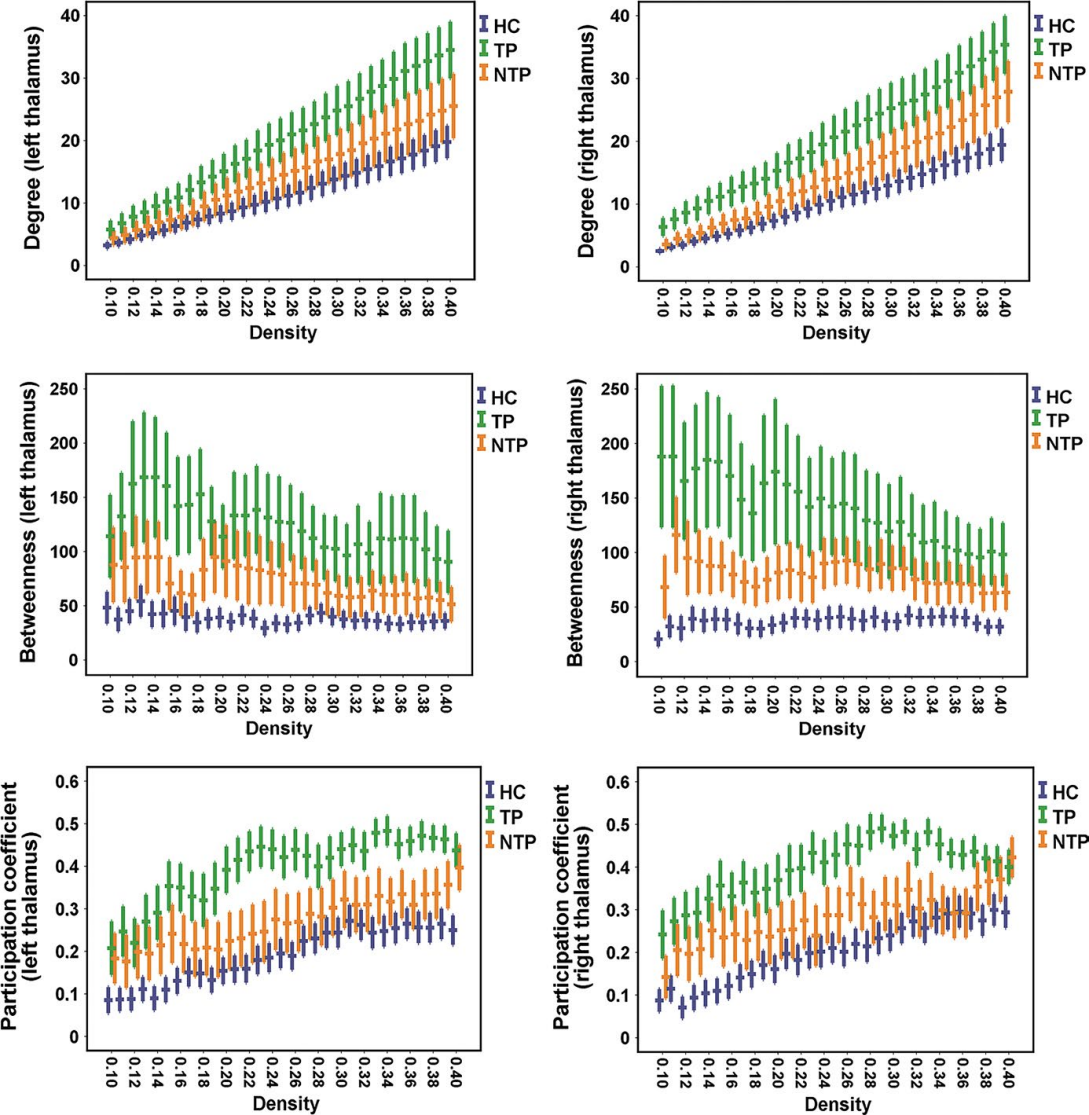
Error plots for centrality metrics of bilateral thalami in patients and controls. The centrality measures were calculated with 31 network densities ranging from 0.10 to 0.40 (*x*‐axis). For each density, the central bands indicate the mean and the error bars indicate the standard errors. Patients with resting tremor (TP group, in green) showed significantly higher values in degree, betweenness, and participation coefficients compared to healthy controls (HC group, in blue); in contrast, no significant differences were shown for these metrics between patients without resting tremor (NTP, in orange) and healthy controls

There was no significant between‐group difference in the modularity estimates (*p *= .14). Three of the four centrality metrics that were measured showed significant main effects in the bilateral thalami across the three groups: degree centrality (*p *= .042 and .010 for the left and right thalami, respectively), betweenness centrality (*p *= .008 and .002 for the left and right thalami, respectively), and participation coefficient (*p *= .001 and .001 for the left and right thalami, respectively). The post hoc *t*‐tests revealed a significant increase in these metrics in the TP group compared to the HC group (degree: *p *= .037 and .010 for the left and right thalami; betweenness: *p *= .006 and .002 for the left and right thalami; and participation coefficient: *p *= .001 and .001 for the left and right thalami, respectively; see Figure 1). In contrast, there were no significant differences in any of these measures in the bilateral thalami between the NTP and HC groups (all *p*s > .39), and between the TP and NTP group (all *p*s > .42). Moreover, no significant main effects were found in the bilateral primary motor cortices or the bilateral cerebella (all *p*s > .05). To ensure that gender distribution did not influence our results, we performed a follow‐up analysis to compare the centrality measures within the male and female subjects separately. Despite great loss of power and thus no significant results were found, the same trends (TP > HC) were also present in both subgroups: the TP group showed trends toward higher centrality measures than HC in both males and females (in males: all *p*s < .20; in females: all *p*s < .50, see Figure S1).

**Figure 2:**
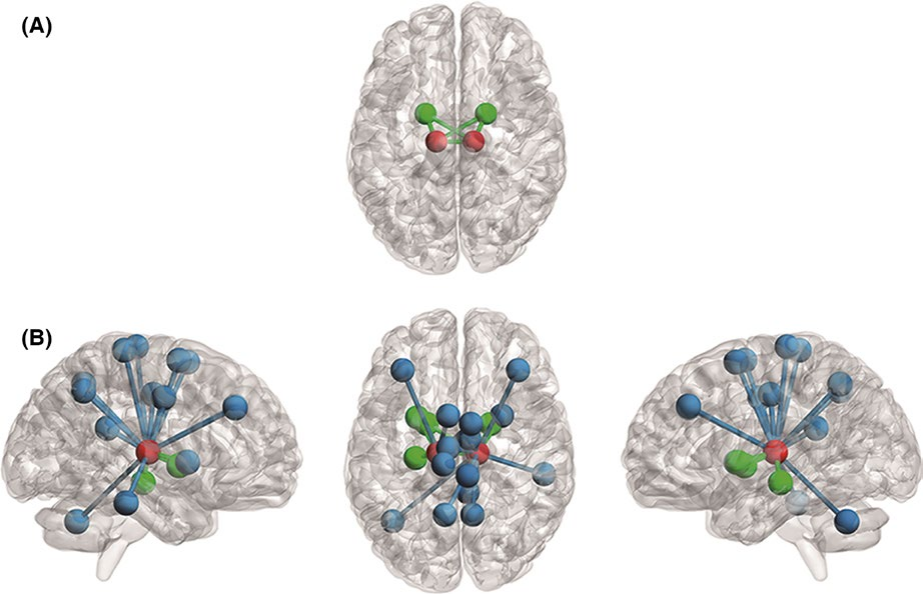
Network illustration for centrality alterations of bilateral thalami in patients with resting tremor. The group networks were calculated by averaging the correlation matrices between all subjects in the respective groups, which were thresholded at a density of 0.30. (A) In the healthy control group (HC), the bilateral thalami (in red) were exclusively connected with the nodes in the same module (bilateral pallida, in green). (B) In patients with resting tremor (TP), there were significantly more connections between the bilateral thalami and nodes in other modules (in blue), especially those involved in the cerebello‐thalamo‐motor cortical loop, including the cerebellum, supplementary motor area, paracentral lobule, and middle frontal gyrus. Details of the nodes and links presented in this figure are listed in Table 3

These results suggest increased importance and intermodular connections of the bilateral thalami in the TP group compared with the HC group. To more precisely probe which intermodular connections were involved in these changes, we further averaged the connectivity matrices for the TP and HC groups separately, thresholded the group matrices at a density of 0.30, and estimated optimal modular structures of the networks for both groups. As shown in Figure 2, in the HC group, the bilateral thalami were exclusively connected with nodes in one module (here the bilateral pallida), while in the TP group, more intermodular connections were identified, including links with the cerebellum, the supplementary motor area, the middle frontal gyrus, the paracentral lobule, the putamen, the cingulate cortex, and the precuneus.

### 3.2 Associations between centrality metrics and tremor severity

**Figure 3:**
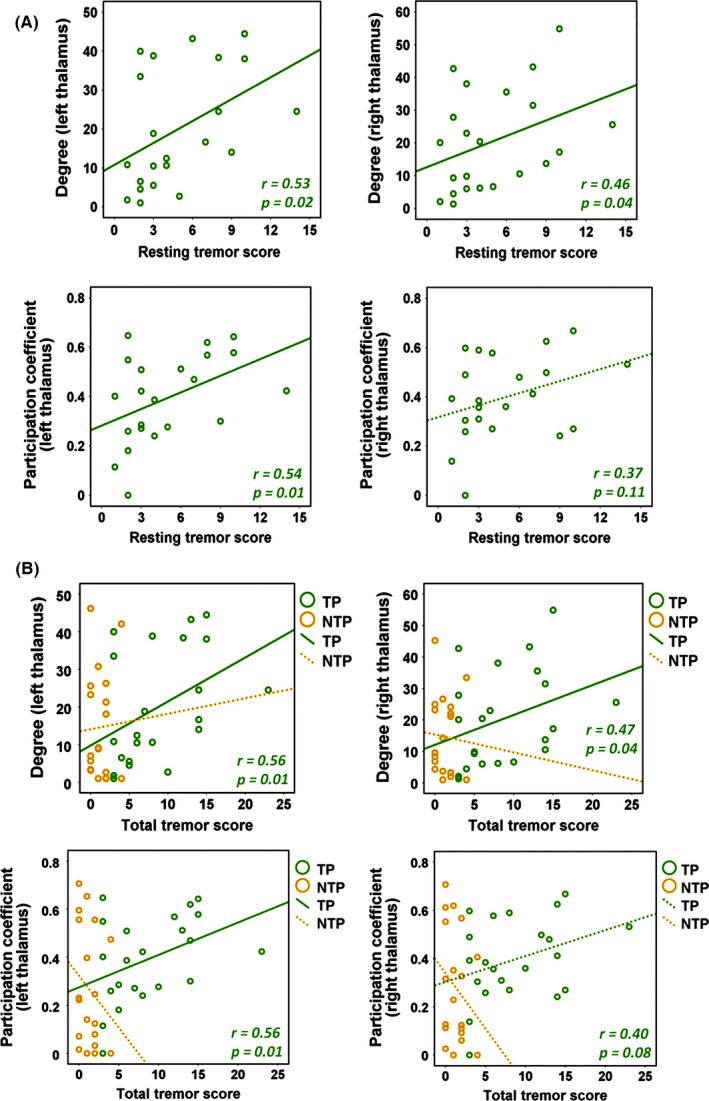
Correlations between centrality measures of the bilateral thalami and UPDRS tremor scores (A: resting tremor scores, B: total tremor scores). In patients with resting tremor (TP, in green), the degree of bilateral thalami and the participation coefficients for the left thalamus were significantly correlated with both tremor scores, and the participation coefficients for the right thalamus showed trend effects. No correlations were shown for patients without resting tremor (NTP, in orange). The solid fitted lines indicate significant effects, and the dashed fitted lines indicate insignificant effects

The partial correlation analyses identified significant associations between the centrality measures and tremor severity in the TP group (Figure 3). Specifically, the degree centrality of the bilateral thalami was positively correlated with the resting tremor scores (*r *= .53, *p *= .02 and *r *= .46, *p *= .04 for the left and right thalami, respectively) and the total tremor scores (*r *= .56, *p *= .01 and *r *= .47, *p *= .04 for the left and right thalami, respectively). Positive correlations were also found for participation coefficients of the left thalamus (*r *= .54, *p *= .01 and *r *= .56, *p *= .01 for the resting and total tremor scores, respectively), and trend effects were found for the right thalamus (*r *= .37, *p *= .11 and *r *= .40, *p *= .08 for the resting and total tremor scores, respectively). In contrast, no significant associations were found for these measures in the NTP group (all *p*s > .33). Further tests for specificity revealed no significant correlations between the centrality metrics and other UPDRS subscores (all *p*s > .28).

### 3.3 Alterations in seed connectivity

**Figure 4:**
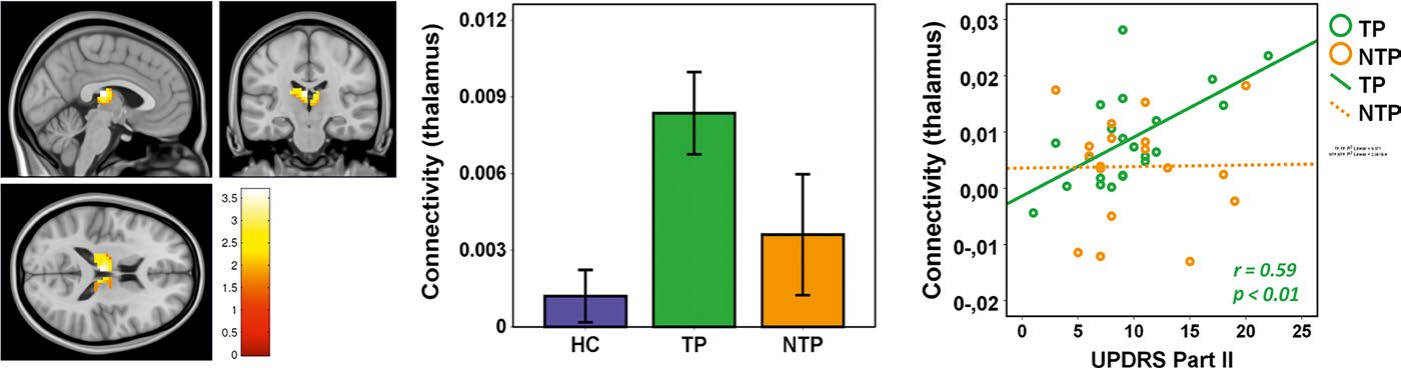
Group‐level contrast map with the putamen as the seed region Left: the group of patients with resting tremor (TP) showed significantly higher putamen–thalamic connectivity compared to the HC group (small‐volume corrected P_FWE_ = 0.027). Middle: patients with resting tremor (TP, in green) showed higher putamen–thalamic connectivity compared to healthy controls (HC, in blue). Right: the altered connectivity was significantly correlated with UPDRS Part II scores (motor assessment of daily life) in the TP group. The statistical significance was set at *p *< .05

The results of the seed‐based analysis are presented in Figure 4, Table 2. With the seed region of the putamen, the TP group showed significantly higher connectivity in the thalamus compared to the HC group (small‐volume corrected P_FWE_ = 0.027), whereas no significant findings were shown in the NTP group compared with the TP group (P_FWE_ = 0.13), and between NTP and HC groups (P_FWE_ = 0.11). Moreover, the analysis for region specificity did not show any significant results for the bilateral cerebella and bilateral primary motor cortices in the TP group (P_FWE_ > 0.81), which suggests that the putamen–thalamic alteration is region‐specific within the CRB‐THA‐MC loop. There were no significant findings between groups for the pallidum as the seed region.

**Table 2:** Brain region with increased connectivity to the putamen in Parkinson's disease patients with resting tremor compared to healthy controls

Anatomical location	Peak coordinates (MNI)			T	Cluster size	Small‐volume P_FWE_ a
	x	y	z			
L. Thalamus	−3	−22	15	3.53	191	0.027

a Small‐volume corrected across mask of the bilateral thalami.

The partial correlation analysis revealed a significant positive correlation between the putamen–thalamic connectivity measures and the UPDRS Part II score (motor aspects of daily living) in the TP group (*r *= .59, *p *= .006). No significant associations were found between the connectivity measures and the tremor scores or other UPDRS scores in either of the patient groups (all *p*s > .16).

## 4 Discussion

This study investigated the hypotheses that parkinsonian resting tremor is associated with alterations in both thalamic centrality and thalamus–basal ganglia functional connectivity. Overall, our results were twofold. First, the TP group showed a significant increase in thalamic centrality compared with the HC group, with more intermodular connections between the thalamus and other brain regions, particularly regions associated with motion. Moreover, the centrality measures in the TP group were strongly related to tremor severity. Second, compared with the HC group, the TP group exhibited enhanced putamen–thalamic functional connectivity, which was positively correlated with daily motor symptoms. These findings provide direct evidence for our hypotheses and highlight the essential role of the thalamus in the pathophysiology of parkinsonian resting tremor.

### 4.1 Centrality alterations in the thalamus

Graph theory analysis employs a set of centrality measures to delineate the importance of a node in the whole‐brain network (Bullmore & Sporns, 2009). With this analysis, we found significantly increased thalamic centrality in the TP group compared to the HC group, particularly in the degree centrality, betweenness centrality, and participation coefficient, thus indicating more intermodular connections and greater importance of the thalamus in the whole‐brain system in the TP group. These findings are highly consistent with previous studies that have shown a strong link between the “hyperfunctional” status of the thalamus and resting tremor (Bergman & Deuschl, 2002; Fukuda et al., 2004; Mehanna et al., 2014; Rehncrona et al., 2003; Wintermark et al., 2014). For example, patients with parkinsonian resting tremor show enhanced thalamic metabolism (Antonini et al., 1998; Kassubek et al., 2001), increased thalamic gray matter volumes (Kassubek, Juengling, Hellwig, Spreer, & Lucking, 2002), and higher thalamic activity (Helmich, Bloem, & Toni, 2011) compared with controls. Here, our results demonstrate that the previously reported “hyperfunctional” status of the thalamus is also present at the level of the whole‐brain network, which leads to increased thalamic centrality. Moreover, our results are strongly supported by a newly refined “finger‐switch‐dimmer” model where the thalamus is the epicenter of PD tremor (Duval et al., 2015). Besides, the group‐level modular patterns showed that the increased intermodular connections in the TP group primarily involved the links with the cerebellum and motor‐related cortices (Figure 2 and Table 1), which supports the current notion that the CRB‐THA‐MC loop is an important regulatory loop for resting tremor.

In contrast to the TP group, we did not detect any significant thalamic centrality alterations in the NTP group. Furthermore, two of the three altered thalamic centrality measures (degree centrality and participation coefficient) correlated positively with the severity of resting tremor rather than other motor symptoms (e.g., bradykinesia, rigidity) in the TP group. These data suggest the thalamic centrality as a potential tremor‐specific imaging measure for patients with PD.

### 4.2 Altered functional connectivity between the CRB‐THA‐MC loop and the basal ganglia

The group‐level modular analysis revealed more connections between the putamen and the thalamus in the TP group (Figure 2 and Table 3). Similarly, the seed‐based connectivity analysis showed enhanced putamen–thalamic functional coupling in the TP group. These results support our second hypothesis and provide further evidence for the previous finding that parkinsonian resting tremor is associated with enhanced interactions between the CRB‐THA‐MC loop and the basal ganglia (Helmich, Janssen, et al., 2011).

**Table 3:** Nodes connected to bilateral thalamus in healthy controls and tremor‐dominant patients, as presented in Figure 2

	L. thalamus	R. thalamus
Healthy controls	R. thalamus	L. thalamus
	L. pallidum	L. pallidum
	R. pallidum	R. pallidum
Tremor patients	R. thalamus	L. thalamus
	L. pallidum	L. pallidum
	R. pallidum	R. pallidum
	L. hippocampus	L. middle cingulate cortex
	L. middle cingulate cortex	R. middle cingulate cortex
	R. middle cingulate cortex	L. cerebellum
	L. middle frontal gyrus	L. precuneus
	L. supplementary motor area	R. precuneus
	R. supplementary motor area	R. middle frontal gyrus
	L. paracentral lobule	R. paracentral lobule
	L. putamen	R. posterior cingulate gyrus
		R. putamen
		R. inferior temporal gyrus
		R. supplementary motor area

L = left; R = right.

In humans, the thalamic intralaminar projection is a major anatomical pathway between the thalamus and the putamen that primarily delivers attention‐related information (Halliday, 2009; Smith et al., 2009). Approximately 30–50% of intralaminar neurons are degenerated in patients with PD, leading to various motor symptoms (Henderson, Carpenter, Cartwright, & Halliday, 2000a,b). Clinical research has shown that stimulation in the intralaminar nuclei is an effective treatment of parkinsonian resting tremor (Krauss, Pohle, Weigel, & Burgunder, 2002; Peppe et al., 2008; Stefani et al., 2009). As a result, the enhanced putamen–thalamic functional connectivity in the TP group may reflect the dysfunctions in the thalamic intralaminar pathway. Moreover, poorer activity performance of daily life was correlated with higher putamen–thalamic functional connectivity in the TP group, which may be due to the overloaded attention inputs in the TP group. Although some studies have argued that pallidal dysfunction leads the CRB‐THA‐MC loop into PD tremor (Bergman et al., 1998; Helmich, Janssen, et al., 2011; Rivlin‐Etzion et al., 2008), a refined brain network model explaining this tremor suggests that the tremor pathology in the pallidum is secondary to the formation of tremor bursting activity in the thalamus and striatum (Duval, Daneault, Hutchison, & Sadikot, 2016). In that brain network model, the thalamus generates the “real” tremor oscillations and PD tremor activity found in the BG nuclei (e.g., Gpi) could therefore simply represent an efferent copy of either thalamic or striatal activity (Duval et al., 2016), which supports our findings that enhanced thalamic centrality and putamen–thalamic functional connectivity were present in the TP group.

It is worth noting that our findings in the TP group do not support some of the prior results reported by Helmich, Janssen, et al. (2011), specifically, the connectivity alterations between the BG nuclei (the putamen and pallidum) and the motor cortex. This incongruence is puzzling, and the reasons can only be speculated on. One possible explanation is the different definitions of seed regions. In our study, we did not document the dominant side of tremor symptoms in patients. Thus, unlike Helmich's study (Helmich, Janssen, et al., 2011), which separated the most‐ and least‐affected hemispheres, we combined both hemispheres in a single analysis. Consequently, our results may reflect an “average” effect across both the most‐ and least‐affected hemispheres in patients with resting tremor. Other possible explanations involve, for example, differences in medication and/or cohort‐specific clinical features. Further studies need to be performed to support these explanations.

### 4.3 Limitations

This study has some limitations. First, our present study only reflected a general relationship between the CRB‐THA‐MC loop and parkinsonian resting tremor, with no effect of hemispheric lateralization on resting tremor has been assessed. Second, as shown in Table 1, there was a significant difference in action tremor scores between the two patient groups. Because resting tremor and action tremor are usually highly interrelated in clinical conditions (Louis et al., 2001; Rana et al., 2014), our results are influenced by this confounding factor. Third, since the CRB‐THA‐MC loop is also involved in other tremor‐related disorders such as essential tremor, which is with overlapping manifestations with parkinsonian resting tremor at initial stages (Cagnan et al., 2014; Nicoletti et al., 2015), the specificity of thalamus for resting tremor still needs further investigation. Finally, the results reported in this study were based on binary networks with multiple thresholds rather than weighted networks, which may affect the partition of the brain networks (Rubinov & Sporns, 2011).

## 5 Conclusions

Using graph theory‐based and seed‐based connectivity analyses, our study demonstrates increased thalamic centrality and enhanced putamen–thalamic functional connectivity in patients with parkinsonian resting tremor. Moreover, the thalamic centrality measures are selectively correlated with tremor severity. Thus, our study suggests the thalamic centrality as a promising tremor‐specific imaging measure for PD and provides evidence for the altered putamen–thalamic interaction in patients with resting tremor.

## Conflict of Interest

The authors have no conflicts of interest to declare.
