# Supplementary material for: Increased thalamic centrality and putamen–thalamic connectivity in patients with parkinsonian resting tremor
Source: Brain Behav. 2016 Nov 23;7(1):e00601. doi: 10.1002/brb3.601 (PMC5256184; doi:10.1002/brb3.601)
Supplement: Supplementary file 1 [file BRB3-7-e00601-s001.docx]

**Supplements**

**Increased thalamic centrality and putamen-thalamic connectivity in patients with parkinsonian resting tremor**

Quanquan Gu, MD, PhD^1, *^; Hengyi Cao, MD, PhD^2, *^; Min Xuan, MD^1^; Wei Luo, MD, PhD^3^; Xiaojun Guan, MD^1^; Peiyu Huang, PhD^1^; Minming Zhang, MD, PhD^1^

1 Department of Radiology, the Second Affiliated Hospital, Zhejiang University School of Medicine, Hangzhou, China

2 Department of Psychiatry and Psychotherapy, Central Institute of Mental Health, University of Heidelberg Medical Faculty Mannheim, Mannheim, Germany

3 Department of Neurology, the Second Affiliated Hospital, Zhejiang University School of Medicine, Hangzhou, China

* These authors contributed equally to this work.

**Supplementary Methods**

In the group consisting of patients with resting tremor (TP), 15 patients were males and 7 females. In the group comprising patients (NTP) in absence of resting tremor, 10 patients were males and 9 females. Of the 45 healthy controls (HC), 24 were males and 21 females.

As illustrated in the Fig.1, the TP group exhibited increased thalamic centrality in measures of degree, betweenness and participation coefficient. To further investigate the possible gender effect on the result, all the patients were reanalyzed based on male/female subgroups separately. The results were displayed below (Fig. S1).


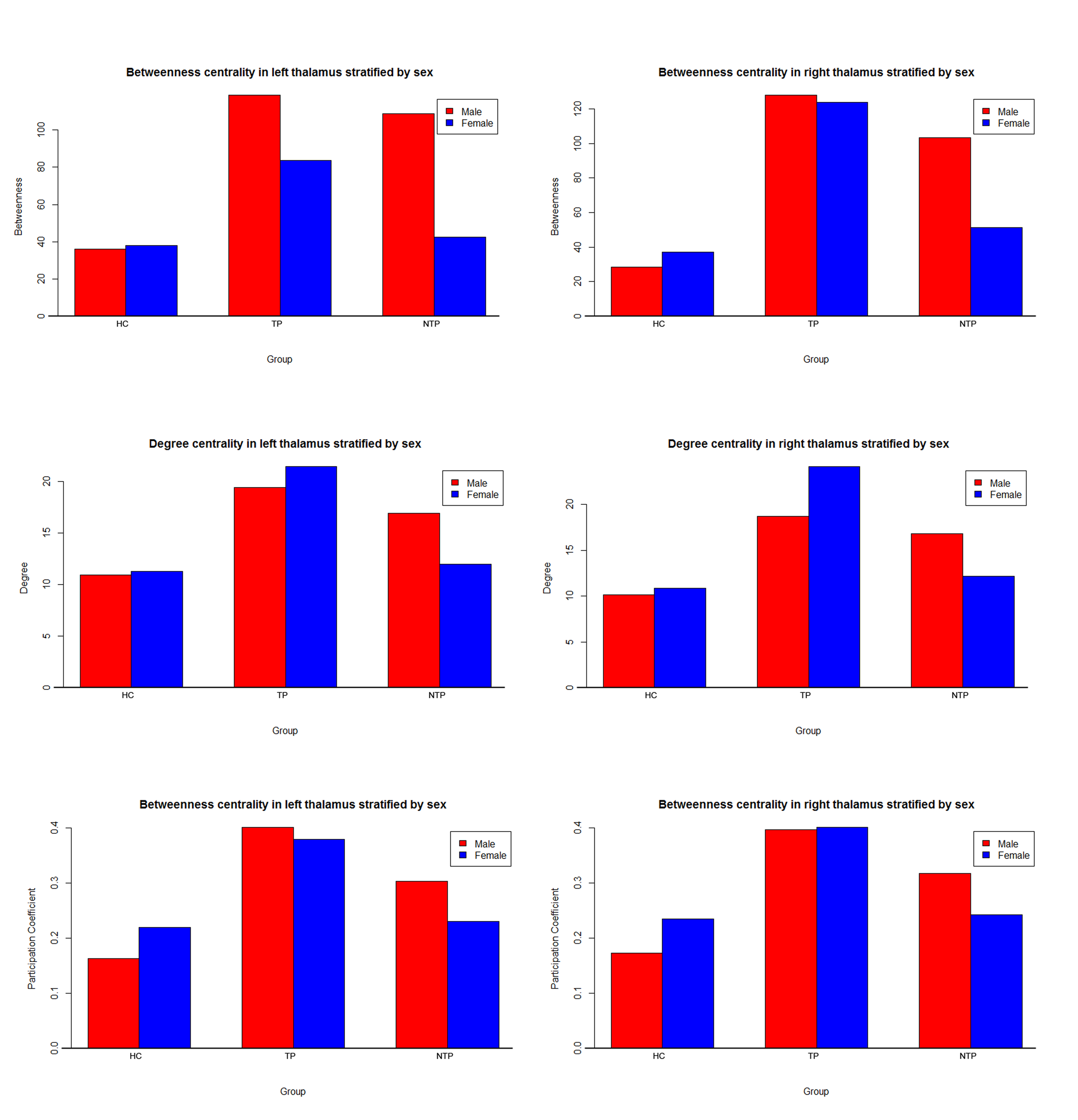


As shown in the Fig. S1, despite the great loss of power and thus no significant results were found, the same trends (TP > HC) were also present in either male or female subgroup: the TP group showed trends towards higher centrality measures (degree, betweenness, and participation coefficient) than the HC group in both males and females (in males: all p values < 0.20; in females: all p values < 0.50).
